# A neuronal reward inequity signal in primate striatum

**DOI:** 10.1152/jn.00321.2015

**Published:** 2015-09-16

**Authors:** Raymundo Báez-Mendoza, Charlotte R. van Coeverden, Wolfram Schultz

**Affiliations:** Department of Physiology, Development and Neuroscience, University of Cambridge, Cambridge, United Kingdom

**Keywords:** inequality, social neuroscience, single neurons, neurophysiology

## Abstract

Primates are social animals, and their survival depends on social interactions with others. Especially important for social interactions and welfare is the observation of rewards obtained by other individuals and the comparison with own reward. The fundamental social decision variable for the comparison process is reward inequity, defined by an asymmetric reward distribution among individuals. An important brain structure for coding reward inequity may be the striatum, a component of the basal ganglia involved in goal-directed behavior. Two rhesus monkeys were seated opposite each other and contacted a touch-sensitive table placed between them to obtain specific magnitudes of reward that were equally or unequally distributed among them. Response times in one of the animals demonstrated differential behavioral sensitivity to reward inequity. A group of neurons in the striatum showed distinct signals reflecting disadvantageous and advantageous reward inequity. These neuronal signals occurred irrespective of, or in conjunction with, own reward coding. These data demonstrate that striatal neurons of macaque monkeys sense the differences between other's and own reward. The neuronal activities are likely to contribute crucial reward information to neuronal mechanisms involved in social interactions.

reward differences between individuals are a part of social life. Individuals regularly compare their reward with others' rewards. Human adults (Adams 1965) and children as young as 3 yr old (LoBue et al. 2011) detect and react to unequal distributions of rewards. Other primates also show reactions to unequal reward distributions. Chimpanzees, macaques, capuchins, and marmosets display differential behavior when faced with unequal reward distributions (Brosnan and De Waal 2003; Burkart et al. 2007; Massen et al. 2010; Proctor et al. 2013). Thus primates are capable of detecting and reacting to reward inequity.

In addition to behavioral characterizations of reward inequity, there is growing interest in brain mechanisms of reward in social situations (Azzi et al. 2012; Báez-Mendoza and Schultz 2013; Chang et al. 2013; Hosokawa and Watanabe 2012). Human neuroimaging studies suggest a role of the striatum in reward inequity (Fliessbach et al. 2007; Harbaugh et al. 2007; Hsu et al. 2008; Moll et al. 2006; Tricomi et al. 2010). For example, neural activity in ventral striatum correlates with the difference between own and other's reward when playing the same skill game (Fliessbach et al. 2007). Overall, it is unknown how individual neurons compare own and other's reward.

Striatal neurons encode own reward value (Apicella et al. 1992; Cai et al. 2011; Cromwell et al. 2005; Cromwell and Schultz 2003; Hikosaka et al. 1989b; Hollerman et al. 1998; Lau and Glimcher 2008; Samejima et al. 2005). This previous work shows a considerable variety of reward-coding neurons in the anterior striatum. Although the mentioned human neuroimaging studies would suggest that individual striatal neurons code reward inequity, this hypothesis has never been tested. Furthermore, few of the neuronal investigations of reward inequity, so far, have differentiated between advantageous and disadvantageous inequity in the same individual. This last point is important, as the sensitivity to disadvantageous and advantageous inequity is dissimilar (Loewenstein et al. 1989). We hypothesized that macaque monkeys were sensitive to the different forms of reward inequity and that striatal neurons would code reward inequity and differentiate between disadvantageous and advantageous inequity. To test these hypotheses, we trained two monkeys, sitting face to face, to take turns to complete a motor task in which the actor and the conspecific received juice rewards. We manipulated the magnitude of these rewards to elicit inequity and differentiate between its disadvantageous and advantageous forms while investigating the activity of striatal neurons in one monkey at a time.

## MATERIALS AND METHODS

### 

#### Animals.

Two adult male rhesus monkeys (*Macaca mulatta*), weighing 9 kg at the start of the experiment, were trained to perform the specific behavioral tasks. The animals were housed together with two other adult male macaques. A third adult male rhesus monkey, weighing 11 kg, received water passively during a supplementary task but was not used for neuronal recordings (monkey C). This animal was housed in a different room with two adult male macaques. All experimental procedures were approved by the UK Home Office under the Animals (Scientific Procedures) Act 1986.

#### Behavioral tasks.

**Fig. 1.**
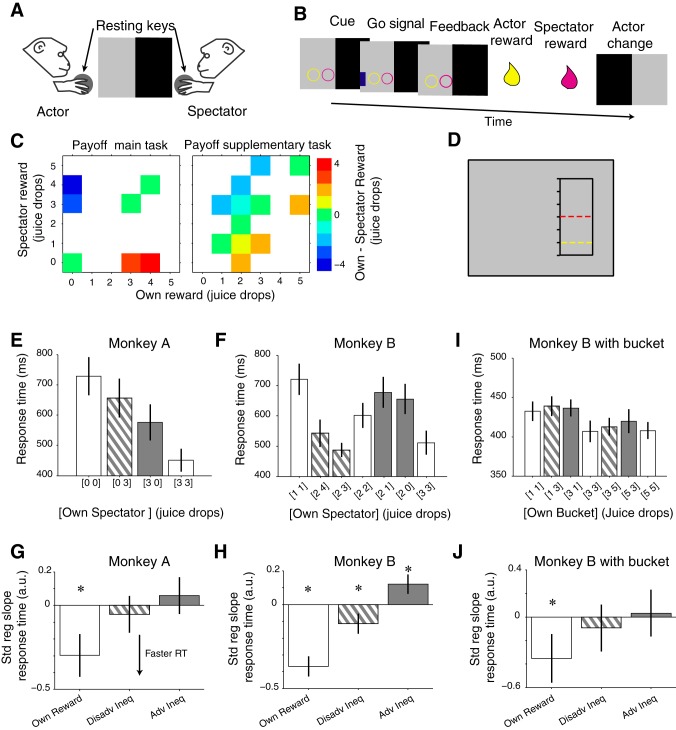
Imperative reward-giving task, reward conditions, and behavioral results. *A*: experimental setup. Two monkeys sat opposite each other at a horizontal computer touch monitor, each contacting a resting key. On each trial, light gray and black backgrounds indicated actor and spectator roles to the respective animals. *B*: task sequence. The shape of the cues predicted absence (square) or presence (circle) of reward for each animal (yellow for left; purple for right animal). The subsequent appearance of a blue Go signal was followed by key release, stimulus touch, and reward for the actor and 1 s later, for the spectator. *C*: reward payoffs in the main (*left*) and supplementary (*right*) tasks for actor and spectator and their corresponding reward inequity. *D*: bar cue used in the supplementary task. The vertical position of each colored bar indicates the number of juice drops for each animal. In the example, “yellow” animal receives 1 juice drop, and “red” animal receives 3 drops. *E*, *F*, and *I*: mean response times (RT) for different reward conditions from 1 experimental trial block completed by monkey A (*E*) and monkey B (*F* and *I*). Monkeys performed the task with a conspecific as spectator (*E* and *F*), and monkey B performed the task with a bucket as a “spectator” (*I*). Error bars show means ± SE. *G*, *H*, and *J*: standardized regression slopes (Std reg slope) for each regressor from the reward inequity model (*Eq. 1*) fit to block mean response times from all experimental trial blocks. Bars show 95% confidence intervals. White bars, own reward; diagonal-striped bars, disadvantageous inequity (Disadv Ineq); gray bars, advantageous inequity (Adv Ineq). *P < 0.05. *E–H*: data were recorded in neuronal inequity test trials.

Task details have been described previously (Báez-Mendoza et al. 2013). Two monkeys sat opposite each other at a horizontally mounted, touch-sensitive computer monitor (Fig. 1*A*) and performed an imperative reward-giving task (Fig. 1*B*). In this task, one monkey was the actor and its conspecific the spectator. The acting monkey moved toward a touchscreen to complete a trial. On a given trial, there was only one option. Thus the animals did not freely choose between options. A color change from black to gray on one-half of the horizontal touch monitor attributed the role of the actor to the corresponding animal. To initiate a trial, the actor contacted a touch-sensitive resting key outside of the monitor for 0.5 s, was presented with a reward-predicting cue for 1 s (composed of circles or a square), waited for a Go signal, and then released the key and touched the Go signal to receive a juice reward (or none, depending on the payoff matrix) after a 2-s delay. The spectator received a reward (or none) 1 s later. In the main task, the role of actor and spectator switched after every trial (monkeys A and B), whereas in the supplementary task, only one monkey (monkey B) was the actor. There were no behavioral requirements for the spectator on either task, other than remaining quiet and abstaining from disruptive behavior. We tested different payoff matrices on each task to assess behavioral and neuronal sensitivities to reward inequity (Fig. 1*C*).

Rewards consisted of blackcurrant juice, made from concentrate, diluted at a ratio of 1:11 by water (Ribena; GlaxoSmithKline, Middlesex, United Kingdom). In the main task, the animals experienced four different reward conditions in a given trial block: reward to neither, only to the recorded animal, only to the spectator, or to both (Fig. 1*C*). The number of cue circles (1–5) indicated the number of juice drops that the specific animal received; each circle predicted 0.2 ml of blackcurrant juice, delivered at 0.15-s intervals. By contrast, unrewarded trials were indicated by a single square. The color of the circles and square specified the monkey receiving this outcome (purple, monkey A; yellow, monkey B; red, monkey C). In the supplementary task, tested on animal B, in addition to the main task, the animal experienced 12 reward conditions from two sets: {[1, 1], [2, 0], [2, 1], [2, 2], [2, 3], [2, 4], [3, 3]} or {[1, 1], [1, 3], [3, 1], [3, 3], [3, 5], [5, 3], [5, 5]} (Fig. 1*C*). In this task, the reward-predicting cue consisted of a rectangle with two bars, in which their vertical positions indicated the number of juice drops and their colors specified the animal receiving these drops (Fig. 1*D*). This cue provided a finer-grained payoff matrix and allowed us to decorrelate the payoff from the number of stimuli shown on the monitor.

During task performance, the actor could commit three different types of errors: *1*) not touching the resting key, *2*) releasing the resting key before the onset of the Go signal, *3*) not touching the touchscreen before a set time (1.4 s). If the actor committed any of these errors, then the screen turned black, and a timeout between 5 and 7 s (remaining trial duration + 0.5 s) ensued, the animals did not switch roles, and the trial was reinitiated.

#### Nonsocial control test.

To test whether monkey B's response time reflected the social nature of the supplementary task, we moved the conspecific animal out of monkey B's sight. The acting animal performed the task as before, but juice delivered to the spectator's side was collected in a transparent bucket. We did not collect neuronal data during these tests.

#### Behavioral data analysis.

Multiple linear regression analysis served to test whether the monkeys' behavior was related to reward inequity. To obtain a measure of behavioral reactivity, we analyzed response time and error rate in relation to own reward and reward inequity using a standard inequity aversion model in which we replaced utility by response time or error rate (Fehr and Schmidt 1999).

(1) $$Y=\beta_{0}+\beta_{1}W+\beta_{2}\max\left(W-Z,0\right)+\beta_{3}max\left(W-Z,0\right)+\varepsilon$$

where *W* = own reward; *Z* = conspecific reward, both in units of juice drops; and *Y* = response time or error rate. We defined response time as the interval between appearance and touch of the Go signal. We defined error rate as the ratio of errors to total trials in each experimental trial block.

The main task design orthogonalized own and conspecific's reward. This allowed us to dissociate behavioral and neuronal responses to own reward from the different forms of inequity. All variance inflation factors as a measure for regressor intercorrelations in the main task were <2.07 and in the supplementary task were <2.092. Thus the regressors of *Eq. 1* showed low intercorrelations.

To compare visually regression coefficients of different magnitudes, we calculated the standardized regression slope. We obtained this by multiplying the regressor by the ratio of the SD of the independent variable (own reward, disadvantageous inequity, or advantageous inequity) to the SD of the dependent variable (response time or discharge rate) (Cai et al. 2011; Kutner et al. 2005).

All statistical tests on behavioral and neuronal data were two tailed unless otherwise noted. All analyses were performed in MATLAB (Mathworks, Natick, MA).

#### Neuronal recording techniques.

Our methods for neurophysiological recording are reported in detail elsewhere (Báez-Mendoza et al. 2013). Briefly, we used conventional electrophysiological techniques to sample extracellular activity from single neurons in the anterior striatum of one monkey at a time, contralateral to the moving arm. We discriminated between slowly discharging neurons and other neurons based on their discharge rates and waveform (Hikosaka et al. 1989a). We isolated the activity of single neurons online using a window discriminator (DIS-1; Bak Electronics, Umatilla, FL) and offline with spike-sorting software (Offline Sorter; Plexon, Dallas, TX). We recorded from the anterior striatum rostral to the anterior commissure, as this region contains reward-sensitive neurons in macaques [e.g., Cai et al. (2011), Cromwell and Schultz (2003), and Lau and Glimcher (2008)].

#### Neuronal inequity coding.

We analyzed neuronal activity during six task epochs of interest, namely: cue onset (between 0.25 s after cue onset and Go-signal onset, 0.75 s later), Go-signal onset (the first 0.5 s after Go-signal onset), movement feedback (0.5 s, starting at Go-signal offset), preceding reward (1.5 s before reward delivery to reward delivery), delivery of own and conspecific's reward (from first juice pulse to 0.5 s later). The time between these several event epochs was fixed, except the interval between Go-signal onset and Go-signal offset, which was determined by response time. We included all recorded trials in our analysis of neuronal inequity coding regardless of the actor (see Movement effort cost below).

To assess relationships between the neuronal activity of each recorded neuron to reward magnitude and inequity, we used the multiple linear regression, defined in *Eq. 1*, in each task epoch, using *Y* for neuronal activity. Only significant fits (F-test, *P* < 0.05) are reported as neuronal modulations. We tested significance for each regression coefficient using a *t*-test (*P* < 0.05). Specifically, a significant slope (*β*_*1*_) for the *W* regressor indicated neuronal coding of own reward. A significant *β*_*2*_ for the *max*(*Z* − *W,0*) term and nonsignificant *β*_*3*_ for the *max*(*W* − *Z,0*) term indicated disadvantageous inequity coding, whereas a significant *β*_*3*_ for the *max*(*W* − *Z,0*) term and nonsignificant *β*_*2*_ for the *max*(*Z* − *W,0*) term indicated advantageous inequity coding. Combinations of significant terms were not uncommon. For example, significant *β*_1_ for the *W* term and significant *β*_*3*_ for the *max*(*W* − *Z,0*) term indicated combined coding of own reward and advantageous inequity. We also obtained partial *R*^*2*^, also called the coefficient of partial determination, for each regressor using standard statistics (Kutner et al. 2005).

Besides own reward and inequity, the total available reward is known to influence activations in human striatum during social interactions (Hsu et al. 2005). A similar assessment in our neurons required inclusion of conspecific's reward magnitude (*Z*) into our analysis, which we considered with the following models:

total available reward

(2) $$Y=\beta_{0}+\beta_{4}\left(W+Z\right)+\varepsilon$$

reward difference between animals

(3) $$Y=\beta_{0}+\beta_{5}\left(W-Z\right)+\varepsilon$$

conspecific's reward magnitude

(4) $$Y=\beta_{0}+\beta_{6}Z+\varepsilon$$

linear combination of own reward magnitude and conspecific's reward magnitude

(5) $$Y=\beta_{0}+\beta_{1}W+\beta_{6}Z+\varepsilon .$$

To identify the best fits among these models, we used the Akaike Information Criterion (AIC), a measure of the fit of non-nested models that provides a measure of the relative strength of evidence for each model in a given set of models. The model with the smallest associated AIC provides the best and most parsimonious fit to the data. The difference in AIC indicates how well the best model performs compared with other models in the set (Lewandowsky and Farrell 2011). To obtain the AIC from general linear models, we used the following equation

(6) AIC=n*log(SSE/n)+2k

where *SSE* is the sum of squared errors, *n* is the number of recorded trials, and *k* is the number of independent variables, including the intercept.

#### Movement effort cost.

The effort required to perform the arm movement constitutes an economic cost that can be subtracted from the reward income and resulted in disadvantageous inequity for the acting monkey when both animals received the same reward amount. With unequal rewards, effort cost would have changed or removed inequity. We considered effort cost when the role of actor changed between the two animals, i.e., during the main task. Therefore, we included a cost term to *Eq. 1* that captured the influence of effort cost by subtraction from the reward for the recorded animal

(7) $$Y=\beta_{0}+\beta_{7}\left(W-c\right)+\beta_{8}max\left(Z-(W-c),0\right)+\beta_{9}max\left((W-c)-Z,0\right)+\varepsilon .$$

We set effort cost to zero when the conspecific was the actor and to nonzero when the recorded monkey acted. We tested several levels of nonzero effort cost to obtain the best fit to inequity-related neuronal activity (*c* = {0.1, 0.3, 0.5, 0.7, 0.9}; *W* and *Z* were set to either 0 for no reward or 1 for reward using the main task only). We then compared best fits between *Eqs. 1* and *7* using the AIC, as described above. We considered that a neuron reflected reward inequity only if the AIC associated with *Eq. 1* was lower than the smallest AIC associated with all effort-cost levels used in *Eq. 7*, suggesting that inequity provided a better fit to the data than any effort cost. By contrast, a neuron may have reflected both reward inequity and effort cost if the AIC associated with *Eq. 1* were higher than the smallest AIC associated with the set of effort-cost levels used in *Eq. 7*, suggesting potential effects of some level of effort cost compromising unanimous assignment to inequity.

#### Response time and neuronal activity.

The striatum contains neurons active during arm movements (Alexander and DeLong 1985). Thus we tested whether the addition of response time to *Eq. 1* provided a better fit to the data

(8) $$Y=\beta_{0}+\beta_{10}W+\beta_{11}max\left(Z-W,0\right)+\beta_{12}max(W-Z,0)+\beta_{13}RT+\varepsilon$$

where *RT* is the response time of the recorded animal and 0 otherwise. Since *Eqs. 8* and *1* are nested, we used a nested F-test to test whether *Eq. 8* provided a better fit to the data than *Eq. 1*.

#### Saccadic eye movement and neuronal activity.

The striatum contains neurons active before and during saccadic eye movement (Hikosaka et al. 1989a). Thus we analyzed whether neurons with inequity-coding activity also reflected task-related saccadic eye movements. We searched for all saccades in the interval between cue onset and actor reward-delivery onset on every trial, binned them into eight cardinal directions, and measured the average firing rate between 250 ms before and 50 ms after each saccade onset in each direction. To estimate saccade direction selectivity, we measured the mean resultant vector length of the vector of mean firing rates for each saccade direction using the CircStat toolbox for MATLAB (Berens 2009). Mean resultant length estimates range from zero, indicating no direction selectivity, to one, indicating perfect saccade direction selectivity. We used a permutation test with 2,000 iterations to test for statistical significance.

#### Histology.

Animals were euthanized with an overdose of sodium pentobarbital (90 mg/kg iv) and perfused with 4% paraformaldehyde in 0.1 M phosphate buffer through the left ventricle of the heart. We introduced marking pins into the brain at previously determined coordinates. Recording positions were reconstructed from 50 μm-thick coronal brain sections stained with cresyl violet.

## RESULTS

### 

#### Behavior.

The regression using *Eq. 1* tested the effects of own reward and inequity and was applied to the means of response times from all trial blocks. It was significant in both animals [animal A: F(3,450) = 9.35, *P* = 5 × 10^−6^, adjusted *R*^2^ = 0.052; animal B: F(3,1271) = 47.45, *P* = 4 × 10^−29^, adjusted *R*^2^ = 0.099]. Example response times from a single inequity test trial block in animals A and B are shown in Fig. 1, *E* and *F*, respectively. The increase in own reward magnitude resulted in shorter response times in both monkeys [Fig. 1, *G* and *H*; animal A: t(450) = −4.62, *P* = 5 × 10^−6^; animal B: t(1271) = −11.91, *P* = 4 × 10^−31^]. Reward inequity significantly affected the response times of monkey B, which were shorter with disadvantageous inequity and longer with advantageous inequity (Fig. 1, *F* and *H*). The increase in disadvantageous inequity shortened response times in both animals, which reached significance in monkey B [Fig. 1, *G* and *H*; animal A: t(450) = −0.943, *P* = 0.34; animal B: t(1271) = −3.79, *P* = 0.0001]. The increase in advantageous inequity prolonged response times in both animals, which reached significance in monkey B [Fig. 1, *G* and *H*; animal A: t(450) = 1.05, *P* = 0.29; animal B: t(1271) = 4.12, *P* = 0.00003].

We also quantified the effect of own reward and reward inequity on error rates. The animals committed an error if they did not touch the resting key at the start of their trial, if they released the resting key before the onset of the Go signal, or if they failed to touch the touchscreen once the Go signal was shown. The acting animal had to repeat the same trial type until completed correctly. Both monkeys made fewer errors the more reward they would obtain [monkey A: t(838) = −4.62, *P* = 4 × 10^−6^; monkey B: t(2232) = −15.74, *P* = 4 × 10^−53^], but only monkey B was sensitive to reward inequity. It made fewer errors with increasing reward inequity in both of its forms [t(2232) < −3.32, *P* < 0.0008]. Together, these results suggest that response times and error rates in one monkey were sensitive to disadvantageous and advantageous inequity.

As a nonsocial control for the effect of reward inequity on response time, we occasionally removed the conspecific during the performance of the supplementary task (Fig. 1*I*). In this control task, monkey B continued to show shorter response times with increasing reward magnitude [Fig. 1*J*; t(126) = −3.36, *P* = 0.001] but now failed to display changes in response times related to reward inequity [Fig. 1*J*; disadvantageous inequity: t(126) = −0.92, *P* = 0.35; advantageous inequity: t(126) = −0.32, *P* = 0.74]. The mean response times of monkey B were shorter during these trials compared with the social situation (446.98 ± 2.06 vs. 525.84 ± 1.31; means ± SE), demonstrating sensitivity to the presence of the conspecific. These results suggest that the effect of reward inequity on monkey B's response time reflected the social nature of the spectator.

#### Overview of neuronal results.

We tested the activity of 152 slowly discharging neurons in the anterior striatum of two monkeys during the performance of the main task (monkey A: *n* = 56 neurons; monkey B: *n* = 96). We previously reported the responses of these neurons in relation to own and conspecific's reward and the social actor (Báez-Mendoza et al. 2013). Neurons from this previous report were not always tested in the condition when neither monkey received a reward, which was not critical for that report, or were tested with an insufficient number of trials for the present analysis. In the present paper, we only included neurons with enough trials on each condition so that we could fit the reward inequity model. Thus here, we report the activity of 152 striatal neurons, whereas we previously reported the activity of 273 striatal neurons. The previously reported reward responses were not related to reward inequity. Of these 152 striatal neurons, 84 (55%) showed 186 reward-related activity modulations during 1 or several of the 6 task epochs and were assigned to 1 of 3 main categories. Specifically, 31 neurons showed 36 modulations reflecting reward inequity without coding the magnitude of the reward to the recorded animal (“own reward”; 11 neurons recorded from monkey A showed 15 modulations); 34 neurons showed 52 modulations reflecting reward inequity together with coding own reward magnitude (16 neurons recorded from monkey A showed 34 modulations); and 60 neurons showed 98 modulations reflecting only own reward magnitude without coding reward inequity (24 neurons recorded from monkey A showed 40 modulations). The number of neurons on each category recorded from each monkey was not significantly different [χ^2^ (2) = 0.93, *P* = 0.62], although the number of modulations on each category was different across animals [χ^2^ (2) = 8.902, *P* = 0.02]. Several neurons showed more than one modulation during the different task epochs and thus adhered to more than one of the three categories.

#### Reward inequity coding without own reward coding.

The activity of 31 of the 152 tested striatal neurons (20%) with 36 modulations signaled specifically the differences in reward between the 2 animals without coding own reward magnitude. The activity of these inequity-coding neurons was significant in the overall regression (*Eq. 1*) and with either the disadvantageous or the advantageous reward inequity regressor or with both inequity regressors. Activity in all of these neurons varied nonsignificantly with the own reward regressor.

Of the 31 striatal neurons, the activity of 18 neurons with 22 modulations reflected disadvantageous inequity without coding own reward (6 neurons and 9 modulations recorded from monkey A). The activity of 12 striatal neurons with 13 neuronal modulations reflected advantageous inequity (5 neurons and 6 modulations from monkey A). The activity of one striatal neuron with one neuronal modulation reflected disadvantageous and advantageous inequity together (recorded from monkey B). Thus neuronal modulations reflecting separately disadvantageous or advantageous inequity were significantly more frequent than modulations coding both inequity forms (35 vs. 1; *P* < 0.001, binomial test).

#### Reward inequity and own reward coding.

**Fig. 2.**
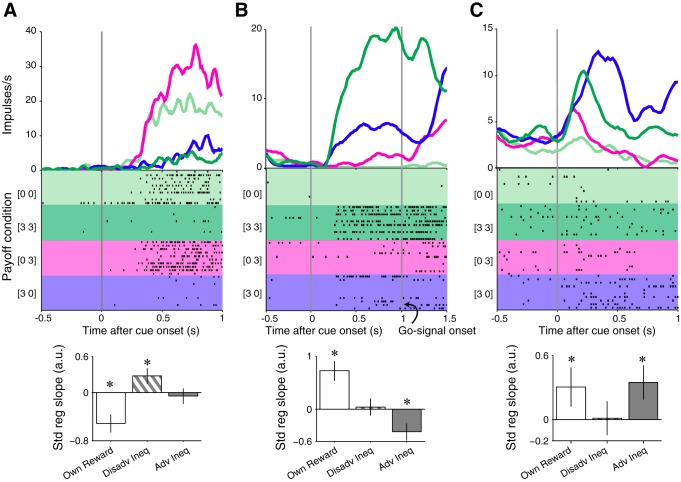
Reward inequity coding in reward-sensitive striatal neurons. *A*: disadvantageous inequity coding in inverse reward magnitude coding neuron, with nonsignificant modulation by advantageous inequity (magenta, disadvantageous inequity; blue, advantageous inequity; green on different shades of green, equity). Note the opposite direction between reward magnitude and disadvantageous inequity coding; recorded from monkey A. *B*: advantageous inequity coding in reward magnitude coding neuron. Note opposite direction between reward magnitude and advantageous inequity coding; recorded from monkey A. *C*: advantageous inequity coding in the same direction as reward magnitude coding; no effect of disadvantageous inequity; recorded from monkey A. *A–C*: rastergrams include only 10 trials of each condition for clarity; bin size = 20 ms. *Bottom*: magnitudes of standardized regression slopes ± 95% confidence intervals for each regressor (*Eq. 1*). *P < 0.05.

The activity of 34 of the 152 tested striatal neurons (22%) with 52 modulations reflected reward inequity and own reward magnitude. Of these 34 striatal neurons, the activity of 17 neurons with 23 modulations reflected disadvantageous inequity (6 neurons and 13 modulations from monkey A), the activity of 17 neurons with 24 modulations reflected advantageous inequity (10 neurons and 16 modulations from monkey A), and the activity of 4 striatal neurons with 5 neuronal modulations reflected disadvantageous and advantageous inequity together (all recorded from monkey A). The number of neurons and modulations on each inequity-related category recorded from each monkey was not significantly different [neurons: χ^2^ (5) = 8.712, *P* = 0.12; modulations: χ^2^ (5) = 8.84, *P* = 0.11]. The large majority of reward inequity modulations with own reward coding was sensitive to only one form of inequity compared with both forms (47 vs. 5, respectively). The activity of the neuron shown in Fig. 2*A* decreased with increasing reward magnitude (green) and increased when the recorded animal received less reward than the conspecific (magenta) but was not modulated when the animal received more reward than the conspecific (blue). Thus this neuron coded disadvantageous inequity in the opposite direction to own reward magnitude coding. The activity of the neuron shown in Fig. 2*B* was lower when the animal received more reward than the other animal (advantageous inequity coding; blue) compared with when both animals received the same reward magnitude (dark green). Thus this neuron coded advantageous inequity in the opposite direction to own reward magnitude coding. The activity of the neuron in Fig. 2*C* increased with advantageous inequity (blue) and with increasing reward magnitude (green). Taken together, the current data suggest that subgroups of neurons in the anterior striatum code specific forms of reward inequity.

#### Effort cost as an alternative to reward inequity.

Movement effort is considered an economic cost (Kagel et al. 1995) that could have asymmetrically reduced the net reward value when only one of the two animals performed a movement, but both animals received a reward. Therefore, we explored the possibility that neuronal activity-coding reward inequity also reflected effort cost. We applied the AIC to select the best-fitting model between *Eqs. 1* (no effort cost) and *7* (additional effort cost; range 10–90%). As a result, the addition of effort cost provided a better fit for only 5 of 46 neuronal modulations that reflected disadvantageous inequity (11%). Similarly, for advantageous inequity, the addition of effort cost provided a better fit for only 7 out of 36 neuronal modulations (19%). Only one out of six neuronal modulations (17%) that reflected both forms of reward inequity benefited from adding effort cost. Taken together, only 15% of neuronal modulations (13/88) that reflected reward inequity could be considered as also being affected by the effort cost of obtaining a reward. These data suggest a relatively small involvement of effort cost in neuronal coding of reward inequity in the recorded striatal neurons.

#### Neuronal results from a supplementary quantitative task.

In the main task, we tested only the contrast between reward presence and absence and therefore, assessed only one level for each type of reward inequity and one level of reward magnitude. To obtain more graded assessments of inequity, we used a supplementary task with several reward magnitudes and accordingly several inequity levels (Fig. 1, *C* and *D*). This task required only one monkey to act, which provided additional opportunities for testing inequity without the potential influence of effort cost. We tested 193 neurons in this task in monkey B.

**Fig. 3.**
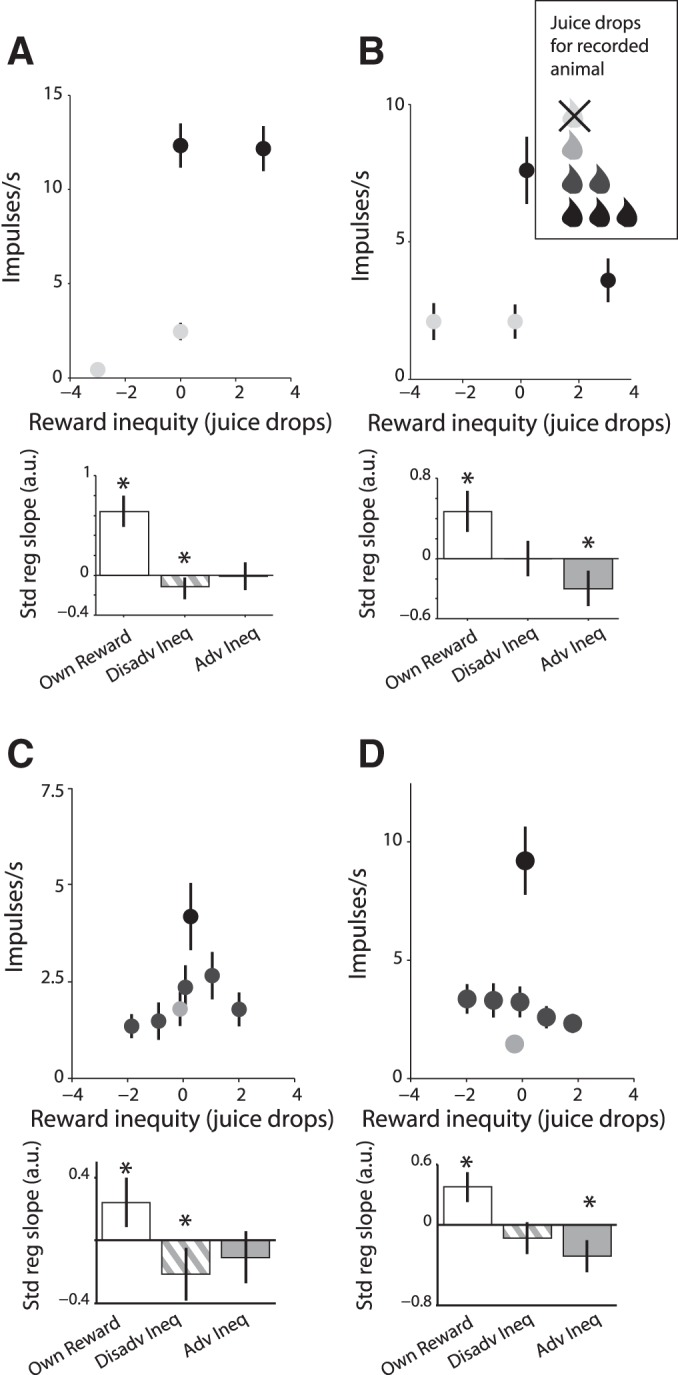
Reward inequity coding in reward-sensitive striatal neurons in different tasks. *A* and *B*: neuronal modulations recorded in the main task showing own reward coding with either disadvantageous inequity (*A*) or advantageous inequity (*B*). Scatter dots show mean neuronal activity in an analysis window. *C* and *D*: same as *A* and *B*, but these neurons were recorded during the supplementary task. In the abscissa, reward inequity is defined as own juice drops minus conspecific's juice drops. Neuronal modulations showing disadvantageous inequity coding demonstrate a decrease in activity with negative reward inequity, and those modulations showing advantageous inequity demonstrate a decrease in activity with positive reward inequity. Scatter dot black saturation indicates the number of juice drops received by the recorded monkey: light gray, 0; medium gray, 1; dark gray, 2; black, 3. Error bars are means ± SE; *P < 0.05.

During performance of this supplementary task, the activity of 90 of 193 tested striatal neurons (47%) showed modulations adhering to one of the three main categories. The activity of 37 of the 193 neurons (19%) reflected reward inequity but not own reward magnitude. Of these, 12 neurons with 15 modulations coded disadvantageous inequity, 17 neurons with 21 modulations coded advantageous inequity, and 9 neurons with 10 modulations coded both forms of reward inequity (1 neuron showed advantageous and both forms of inequity coding with different task events). The activity of 19 of the 193 neurons (10%) reflected reward inequity and own reward magnitude. Of these, 7 neurons (8 modulations) coded disadvantageous inequity, 10 neurons (12 modulations) coded advantageous inequity, and 2 neurons (2 modulations) coded both forms of inequity. These results from the supplementary task confirm the inequity coding seen in the main task and provide more quantitative information about inequity coding (Fig. 3). In this figure, reward inequity was defined as own juice drops minus conspecific's juice drops. Thus disadvantageous inequity equals negative reward inequity, and advantageous inequity equals positive reward inequity. The activity of 54 of the 193 neurons (28%) with 65 modulations coded own reward magnitude irrespective of reward inequity. The number of neurons in each category varied nonsignificantly between the main and supplementary tasks [χ^2^ (2) = 4.15, *P* = 0.12]. Thus the supplementary task with extended payoff matrix and constant effort cost confirmed all main types of reward-related coding found in the main task. In the following analyzes, we pooled the data from both tasks. They are summarized in Table 1.

**Table 1.** Number of modulations (neurons) coding reward inequity

	Disadvantageous Inequity		Advantageous Inequity		Both Forms of Inequity		Subtotal	
No reward magnitude	37	(30)	34	(29)	11	(10)	82	(68)
Reward magnitude	32	(24)	35	(27)	7	(6)	74	(53)
Subtotal	69	(50)	69	(53)	18	(16)	156	(111)

Numbers of single neuronal modulations with at least 1 significant modulation during 1 task period. Some neurons (numbers in parentheses) showed effects in multiple periods, sometimes coding 1 form of reward inequity during 1 period and another form of reward inequity in a different period. Therefore, the number of neurons in subtotal rows and columns may be lower than the sum of neurons in respective individual rows or columns.

#### Direction of reward inequity coding.

**Fig. 4.**
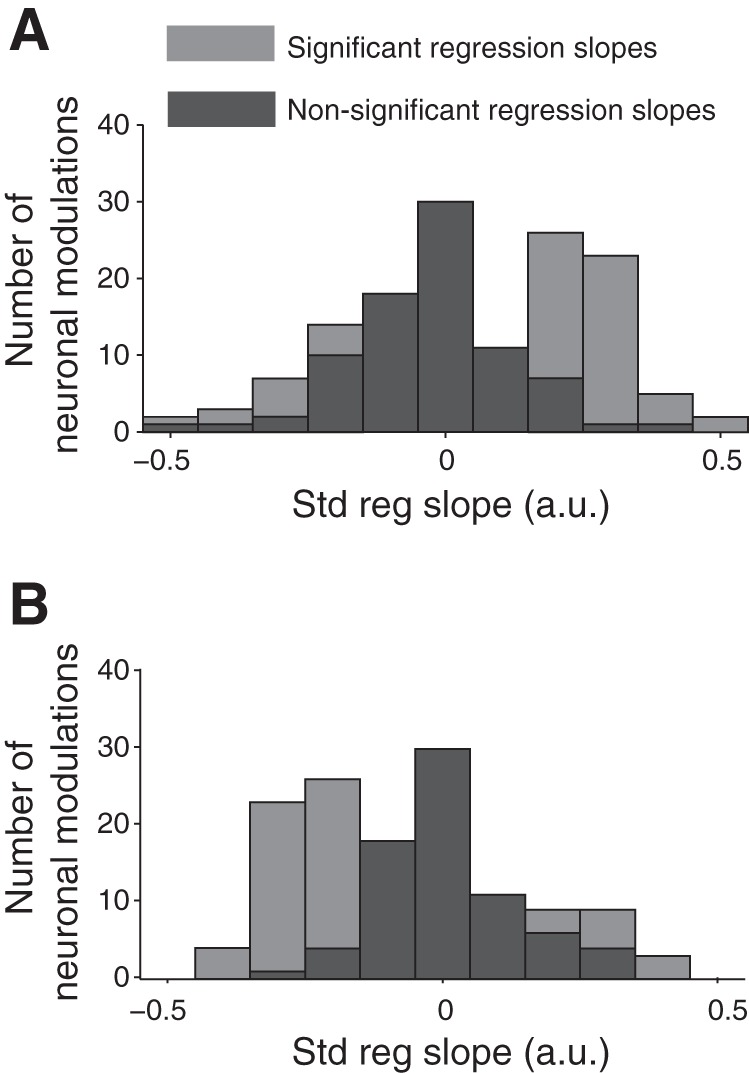
Standardized regression slopes of reward inequity coding. *A*: neuronal modulations not coding own reward magnitude. *B*: neuronal modulations also coding own reward magnitude. Gray, significant regression slopes; black, nonsignificant slopes.

The neurons coding reward inequity showed increasing or decreasing activity with increasing inequity, as documented by positive or negative regression slopes, respectively (Fig. 4). Neurons coding inequity, irrespective of own reward, showed a predominantly positive regression slope for inequity (53 modulations with a positive sign out of 71, *P* = 0.00004, binomial test; Fig. 4*A*). By contrast, neurons coding reward inequity and own reward magnitude were more likely to be associated with a negative regression slope (46 modulations with a negative sign out of 67, *P* = 0.003, binomial test; Fig. 4*B*). Thus neuronal modulations that only coded reward inequity were more likely to reflect reward inequity with an increase in discharge rate rather than a decrease. By contrast, neurons coding own reward magnitude together with reward inequity more likely reflected reward inequity by a decrease in discharge rate rather than an increase. Thus the direction of coding (activity increase or decrease) depended on whether the neuron's activity reflected own reward magnitude.

#### Comparison between inequity and own reward coding.

To compare reward inequity coding with own reward coding, we estimated the partial *R*^2^ on neurons coding both inequity and own reward magnitude. Regressions of neuronal responses to disadvantageous inequity showed a lower partial *R*^2^ than regressions on own reward magnitude (0.05 ± 0.004 vs. 0.095 ± 0.015; means ± SE). Similarly, neuronal coding for advantageous inequity was associated with a lower partial *R*^2^ than for own reward (0.07 ± 0.005 vs. 0.118 ± 0.015; means ± SE). Thus although the inability to set comparable scales precludes more firm conclusions, inequity coding might be weaker than own reward coding.

#### Task epoch distribution.

**Fig. 5.**
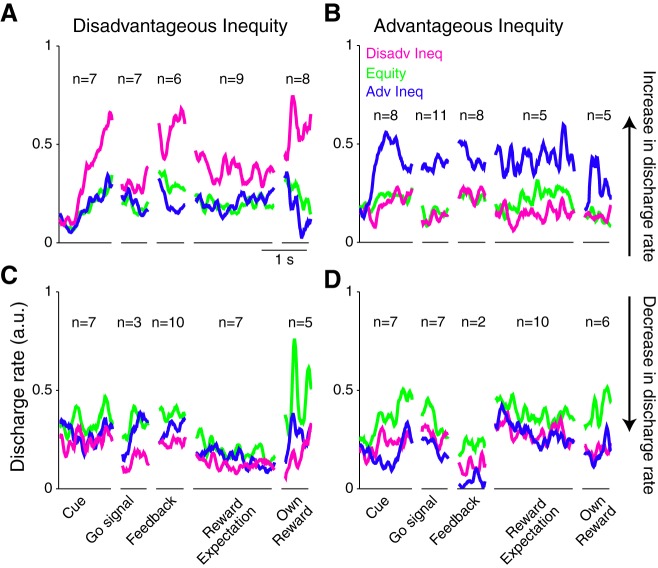
Distinct reward inequity coding in neuronal population activity. *A*: normalized spike density function (SDF) of neurons within individual trial epochs showing significantly higher discharge rates with increasing disadvantageous inequity (magenta) compared with equal rewards (green) and advantageous inequity (blue); *n* = 37. *B*: population SDF of neurons showing significantly higher discharge rates with increasing advantageous inequity; *n* = 37. *C* and *D*: population SDF of neurons within 1 trial epoch showing significant decreases in discharge rate with either disadvantageous (*C*; *n* = 32) or advantageous (*D*; *n* = 32) reward inequity.

Inequity neurons showed significant modulations during all analyzed task epochs (Fig. 5). Neuronal activity increased (Fig. 5, *A* and *B*) or decreased (Fig. 5, *C* and *D*) in relation to disadvantageous inequity (Fig. 5, *A* and *C*) or advantageous inequity (Fig. 5, *B* and *D*). The difference in the number of modulations in each category and each epoch was nonsignificant [χ^2^ (12) = 12.55, *P* = 0.4; Fig. 5]. These data suggest that anterior striatal neurons code reward inequity throughout the entire trial.

#### Anatomical distribution.

**Fig. 6.**
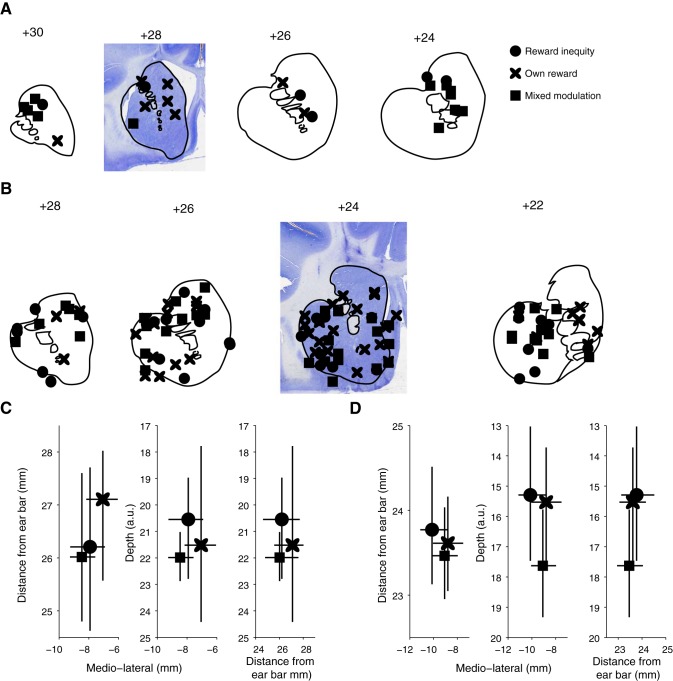
Neuronal recording locations. *A*: coronal series showing the recording locations in monkey A for reward-modulated striatal neurons, plotted separately for each neuronal category: modulation by reward inequity (circles), own reward (crosses), and mixed (rectangles). Striatum outline was drawn from the atlas of Saleem and Logothetis (2007). The background in section +28 is a Nissl-stained brain coronal slice from monkey A, used for recording position reconstruction. *B*: coronal series showing the recording locations in monkey B; conventions as in *A*. The background in section +24 is a Nissl-stained coronal slice from monkey B. Note gliosis in white matter dorsal to the caudate. *C* and *D*: centroids (mean position in all coordinate directions) of each class of neurons recorded in monkeys A and B, respectively. Error bars are 95% confidence interval, estimated with bootstrap (20,000 iterations).

Figure 6, *A* and *B*, shows the location of striatal neurons that coded reward inequity only, own reward only, or both. These neurons were distributed over a large area in the striatum, without clustering in a particular region. To investigate the regional distribution, we obtained the centroids (the mean position in all of the coordinate directions) of each class of neurons and estimated a 95% confidence interval with bootstrap (20,000 iterations). We hypothesized that if a particular class of neurons clusters away from other classes, then its confidence interval should not overlap with that of other classes. We observed that all confidence intervals overlapped from each neuronal class on each dimension (anterior-posterior, mediolateral, and depth) and on each animal (Fig. 6, *C* and *D*). These results suggest that within our sample, striatal neurons modulated by reward inequity, own reward, or both overlapped in their anatomical position in the striatum.

#### Alternatives to reward inequity coding.

Neuronal activity, classified as reflecting reward inequity, might be explained by reward processes unrelated to inequity. We considered four alternative hypotheses; the hypotheses were associated with a single linear regression (total available reward, *Eq. 2*; reward difference, *Eq. 3*; and conspecific's reward, *Eq. 4*) or with a multiple linear regression (own reward and conspecific's reward, *Eq. 5*). To decide which model provided the best fit to the data, we compared the goodness of fit of all models using the AIC (*Eq. 6*). The large majority of neuronal inequity modulations was best fit by the reward inequity model (126 out of 156; 81%). Of the remaining 30 neuronal modulations, 10 were better explained by conspecific's reward, 10 reflected the sum of available reward, and 10 coded the 2 forms of inequity in opposite directions, which amounted to a continuous coding of reward difference between the recipients and was not considered further. No neuronal modulation was better explained by the linear combination of own reward magnitude and conspecific's reward magnitude. Thus only a minority of modulations, classified as coding reward inequity, might have reflected other reward processes.

Neuronal activity in the striatum is related to limb movement (Alexander and DeLong 1985). Therefore, it is possible that neuronal modulations reflecting reward inequity and own reward also coded movement parameters. To account for this possibility, we added a term for response time to *Eq. 1* and tested whether this model (*Eq. 8*) provided a better fit than the original *Eq. 1* using a nested F-test. Indeed, 76 of the 186 neuronal modulations recorded in the main task (41%; 50 of 89 neuronal modulations recorded in monkey A) were better fit by *Eq. 8* than *Eq. 1*. Similarly, 20 of the 133 neuronal modulations recorded in the supplementary task (15%) were better fit by taking into consideration arm movements. These data suggest that a fraction of neuronal modulations related to reward inequity was also modulated by arm movements.

Striatal neurons are also involved in saccadic eye movements (Hikosaka et al. 1989a). Therefore, inequity-coding neurons might also show relationships to eye movements. To test this, we determined whether a neuron showed significant task-related saccade direction selectivity using a permutation test on the mean resultant vector, indicating strongest directional selectivity. A total 12 of 115 striatal neurons recorded alongside eye monitoring showed task-related saccade direction selectivity. Of the neurons that showed saccade direction selectivity, only five also contained reward inequity modulations (out of 73 reward inequity-coding neurons that were recorded alongside eye position of the recorded animal). Thus very few inequity-coding neurons were also involved in saccadic eye movements.

## DISCUSSION

These data suggest that some striatal neurons are sensitive to reward inequity. The behavioral results provided evidence for moderate inequity sensitivity in the laboratory setting. The striatal neurons coded either disadvantageous inequity (receiving less reward than the conspecific) or advantageous inequity (getting more reward than the conspecific), although a few coded both inequity forms. A sizable fraction (44%) of striatal inequity neurons also coded own reward magnitude. Control analyses largely ruled out confounding task relationships, including total reward for both animals, conspecific's reward, simple monotonic reward difference between animals, linear combination of own and conspecific's reward, effort cost, and eye movements. These inequity-coding responses differed from the previously reported responses in striatal neurons that coded mostly own reward, irrespective of the conspecific's reward (Báez-Mendoza et al. 2013); only some of these striatal neurons also showed responses reflecting reward inequity. Taken together, the present results suggest that a population of striatal neurons signals the inequity between own and others' reward.

Previous and present behavioral results suggest that rhesus monkeys may be sensitive to reward for other monkeys (Azzi et al. 2012; Chang et al. 2011). One monkey's response times decreased with increasing disadvantageous inequity and increased with increasing advantageous inequity, and both animals' response times were shorter with increasing own reward magnitudes, confirming earlier results (Watanabe et al. 2001). Furthermore, the limited social control tests suggested that the inequity-related changes in behavioral response times required the presence of a biological agent (rather than a transparent bucket). However, these behavioral results do not reveal in an unequivocal manner the influence of reward inequity on subjective reward value, as demonstrated and formalized for humans before (Fehr and Schmidt 1999; Loewenstein et al. 1989). Nevertheless, the behavioral sensitivity to reward inequity appeared to be driven by the social nature of the task.

Macaque monkeys live in troops with hierarchies (Maestripieri 2008), opening the possibility that the coding of reward inequity can be modulated by differences in social status. In our task, monkey A ranked higher than monkey B (Báez-Mendoza et al. 2013), which might be related to its lack of behavioral sensitivity to reward inequity. However, two animals produced too few data points to draw significant conclusions about the role of hierarchy on behavioral sensitivity to reward inequity.

It is possible that rather than being socially motivated, the results can be explained by a combination of selfish motives. On one hand, fear of reprisal by the partner for getting more reward might explain the slower response times in advantageous inequity situations. On the other hand, a desire to get past the current trial and move on to the next one might explain the faster response times with disadvantageous inequity. Whether rhesus monkeys behave in this type of tasks with a social motive or a selfish motive remains to be tested.

Most of our tested striatal neurons reflected only one form of reward inequity. Thus these striatal neurons did not code the absolute difference in reward; otherwise, they would reflect both disadvantageous and advantageous reward inequity. Rather, these neurons provided a signed comparison of the rewards between animals. In support of this interpretation, Tricomi and colleagues (2010) report that activations in the human striatum reflected one form of reward inequity depending on the relative wealth of the individuals. Interestingly, there are only few striatal neurons coding conspecific's reward (Báez-Mendoza et al. 2013), which may suggest that the reward inequity signal is computed elsewhere and transmitted “as is” to the striatum. One candidate area for this computation might be the prefrontal cortex (PFC). Neurons in this area encode unidirectional contrasts (e.g., greater than or smaller than) between numerosities (Bongard and Nieder 2010) and differentiate between reward outcomes in a social competition task (Hosokawa and Watanabe 2012). Furthermore, many neurons in this area project to the striatum (Haber and Knutson 2010). Another neuronal substrate of conspecific reward coding might lie in the anterior cingulate cortex gyrus (ACC), as single neurons there respond to reward delivered to the conspecific (Chang et al. 2013). Information about the conspecific's reward might be transmitted directly from the ACC gyrus to PFC, including orbitofrontal cortex (OFC) (Pandya et al. 1981). In support of this interpretation, closer inspection of the published data reveals that value-coding neurons in OFC decrease their discharge rate if a conspecific will also receive a reward (Azzi et al. 2012). In conclusion, different forms of inequity might be coded by different subpopulations of neurons in PFC that transmit this information to the striatum.

We compared the neuronal coding strength between reward inequity and own reward. With the reservations imposed by the lack of a common scale, we noted that coding of reward inequity was smaller than that of own reward. These data might suggest that inequity coding was supplementary to the coding of the currently expected reward, an effect expected in neurons that integrate inequity into own reward magnitude coding. Furthermore, the weaker inequity coding raises the possibility that the hypothesized inequity signal coming from prefrontal neurons is added to the own reward signal present in striatal neurons. Concurrent measurement of neuronal activity in both of these brain regions, possibly on a common scale between inequity and own reward, could yield insights into this question.

Striatal neurons are known to be engaged in the processing of a wide range of different task events throughout whole experimental trials. These neurons show relationships to visual cues, movement preparation and execution, and expectation and delivery of reward (Kim et al. 2009; Schultz et al. 1998; Seo et al. 2012). Thus it was interesting to observe that inequity-related neuronal modulations occurred throughout the whole trial. Although the animals only needed to compute reward inequity one time, once the cues were shown, the neuronal signal remained present. Thus the signal allowed the brain to continue computations and maintain representations of reward inequity, where it could influence other kinds of neuronal activity.

The activity of inequity-coding neurons could alternatively be explained by the magnitude of available reward or by reward difference. Previous neuroimaging studies have shown that ventral striatum activity was related to reward difference (Fliessbach et al. 2007) and to both the inequity and the total reward to all participants (Hsu et al. 2008). We considered both of these possibilities using single linear regressions. However, neither model, accounting for reward difference or total magnitude of available reward, provided a better fit to the data than the inequity model in the vast majority of neurons. Furthermore, most striatal neurons reflected only one form of reward inequity, which suggests that they do not code the absolute difference in reward. These results suggest that neither total reward to all participants nor reward differences were good models of the current neuronal data.

Differences in the effort cost to obtain a reward can affect reward inequity (Adams 1965; Kagel et al. 1995). In the main task, the recorded monkey spent effort when it moved but no effort when the conspecific completed a trial. We used these two effort-cost levels to analyze the effect of effort cost on neuronal coding of own reward magnitude and reward inequity. Only a minority of neurons (15%) that reflected reward inequity was also modulated by effort cost. This result suggests that reward inequity coding during the main task was marginally affected by differences in the effort cost to acquire rewards. In the supplementary task, the effort cost to execute the task was only paid by the recorded monkey. This situation might give rise to effort-cost inequity. However, the animal paid this effort cost regardless of reward inequity. Therefore, any effort-cost inequities in this task can be assumed to be constant, thus excluding it as an explanatory factor in our design. The role of effort-cost inequity in value coding and its neuronal basis is a separate issue that remains to be explored.

We note that we lack a nonsocial control experiment to test whether inequity-sensitive neurons were sensitive to the social context. It could be that inequity neurons responded to the fact that more (or less) reward was delivered to the partner side but not to the fact that another monkey got the other reward. Future experiments could disambiguate the role of the social context in the responses to inequity by striatal neurons.

Different regions of the striatum receive differential input from prefrontal cortical areas (Haber and Knutson 2010). It is believed that neurons located in these different regions may form part of different cortico-basal ganglia loops (Alexander et al. 1986). Our recording reconstruction suggests that we targeted neurons located in the anterior striatum and mostly—but not exclusively—the caudate and putamen. Our recordings probably encompassed several of these loops. However, we did not find clustering of the main types of neurons in any region. These results suggest that striatal neurons coding reward inequity are widely distributed across the anterior striatum.

As the striatum is an entry node to the basal ganglia, it is reasonable to assume that the reward inequity signals reported here influence the activity of downstream structures, including dopaminergic cells. Dopaminergic cells reflect in their reward prediction error responses the economic values of different goods and their marginal utility (Lak et al. 2014; Stauffer et al. 2014). Thus the reward inequity information transmitted by striatal cells could potentially modify the reward prediction error signal generated by dopaminergic cells. This modified reward prediction error signal could update economic value coding elsewhere in the brain, including the frontal areas that form part of the cortico-basal ganglia loops. However, it remains to be tested if reward inequity can modify subjective utility in monkeys.

In humans, reward comparisons change the subjective value of a reward for oneself (Bolton and Ockenfels 2000; Fehr and Schmidt 1999; Loewenstein et al. 1989). In the wild, rhesus macaques strive to obtain better rewards by attempting to climb in their social hierarchy (Maestripieri 2008). In our experiment, one monkey responded slower when it would obtain more reward than the conspecific and faster when the conspecific would obtain more reward. By manipulating disadvantageous and advantageous inequity along with reward value in the same experimental task, we were able to probe the neuronal correlates of reward inequity in value-coding neurons. Indeed, some of the observed striatal neurons coded both reward magnitude and inequity. An organism embedded in a social environment equipped with such a neuronal mechanism would not only be able to detect the rewards that others receive but also could compare other's reward with what it already has. Armed with this knowledge, an organism will be able to select more valuable actions for itself.

## GRANTS

Support for this research was provided by the Wellcome Trust, European Research Council (ERC), and NIH Conte Center at the California Institute of Technology (Pasadena, CA).

## DISCLOSURES

No conflicts of interest, financial or otherwise, are declared by the authors.

## AUTHOR CONTRIBUTIONS

Author contributions: R.B-M. and W.S. conception and design of research; R.B-M. and C.R.v.C. performed experiments; R.B-M. analyzed data; R.B-M. and W.S. interpreted results of experiments; R.B-M. prepared figures; R.B-M. drafted manuscript; R.B-M. and W.S. edited and revised manuscript; R.B-M., C.R.v.C., and W.S. approved final version of manuscript.
